# Robotic-assisted laparoscopic retrieval of a migrated IUCD in the pelvis

**DOI:** 10.1093/jscr/rjab268

**Published:** 2021-07-05

**Authors:** Kelvin Adasonla, Mohammed K Quraishi, Abdoulai Samateh, Rebecca Tregunna, Edward Calleja, Peter D Rimington

**Affiliations:** Department of Urology, Eastbourne District General Hospital, Eastbourne, UK; Department of Urology, Eastbourne District General Hospital, Eastbourne, UK; Department of Urology, Eastbourne District General Hospital, Eastbourne, UK; Department of Urology, Eastbourne District General Hospital, Eastbourne, UK; Department of Urology, Eastbourne District General Hospital, Eastbourne, UK; Department of Urology, Eastbourne District General Hospital, Eastbourne, UK

## Abstract

Intrauterine contraceptive devices (IUCDs) are a popular treatment choice for contraception. We report a case of a woman in her forties who presented to a urology clinic with visible haematuria. Flexible cystoscopy revealed a bladder lesion, suspicious for a tumour. However, subsequent imaging determined that this was in fact the arm of an IUCD, sited 7 years previously, which had migrated into the bladder. The patient underwent an uneventful robotic-assisted laparoscopic removal of the device.

IUCD-related complications are infrequent and can present atypically, warranting a broad diagnostic approach. Robotic-assisted laparoscopic removal of devices migrating into pelvic structures offers all the advantages of minimally invasive surgery, with the added benefits of three-dimensional views and endowrist movement facilitating tasks such as intracorporeal suturing. We report the first documented case of utilizing the da Vinci robotic system in safely assisting the removal of a migrated IUCD.

## INTRODUCTION

Intrauterine contraceptive devices (IUCDs) are an effective contraception option, popular worldwide [1]. Associated complications include uterine perforation, documented in 0.2% of cases [2]. Although migration of IUCD into surrounding tissues is rare, devices have been located in adjacent abdomino-pelvic structures [3]. Presentation of IUCD migration is variable, depending on anatomical location. Here, we report our experience of robotic-assisted device retrieval.

## CASE REPORT

### Case presentation

A 42-year-old woman presented to our urological clinic through our ‘Two Week Wait’ suspected malignancy pathway. She reported several episodes of painful visible haematuria over several weeks.

Her past surgical history included two caesarean sections, and her medical history was otherwise unremarkable. She had a Copper TT380 slimline IUCD (Durbin PLC, Hayes, UK) inserted 7 years previously, several months after the birth of her youngest child.

### Investigations

**Figure 1:**
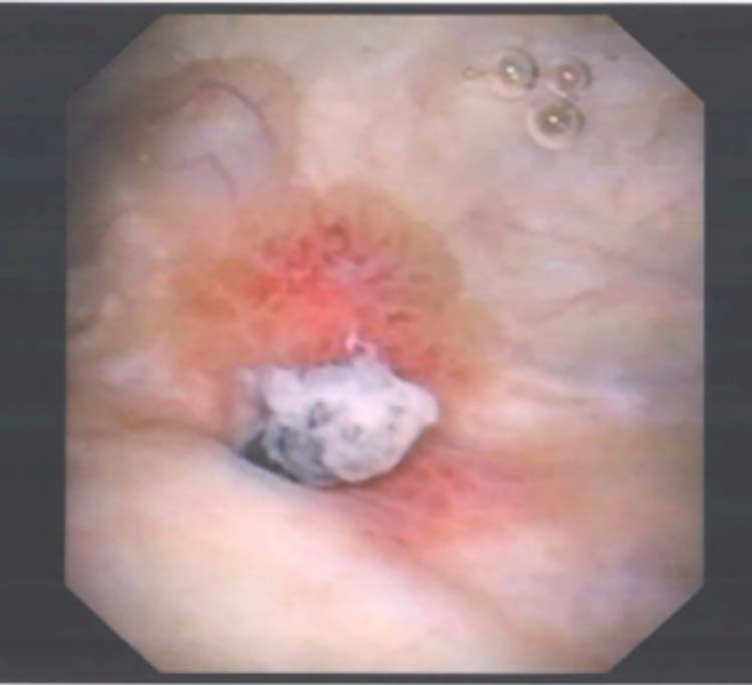
Image of suspected ‘tumour’ on right posterolateral wall of the bladder, taken during flexible cystoscopy.

Mid-stream urine culture was negative. Flexible cystoscopy identified a 1 cm raised abnormal lesion on the right postero-lateral wall, suspicious for malignancy (Fig. 1).

**Figure 2:**
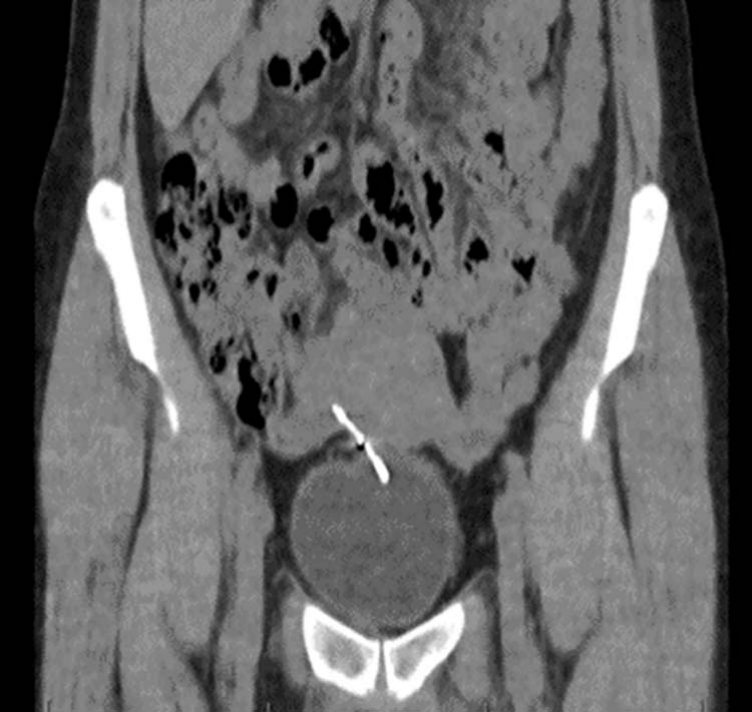
Coronal CT urinary tract image demonstrating the device lying between uterus and bladder, with one horn of the device embedded into the bladder.

**Figure 3:**
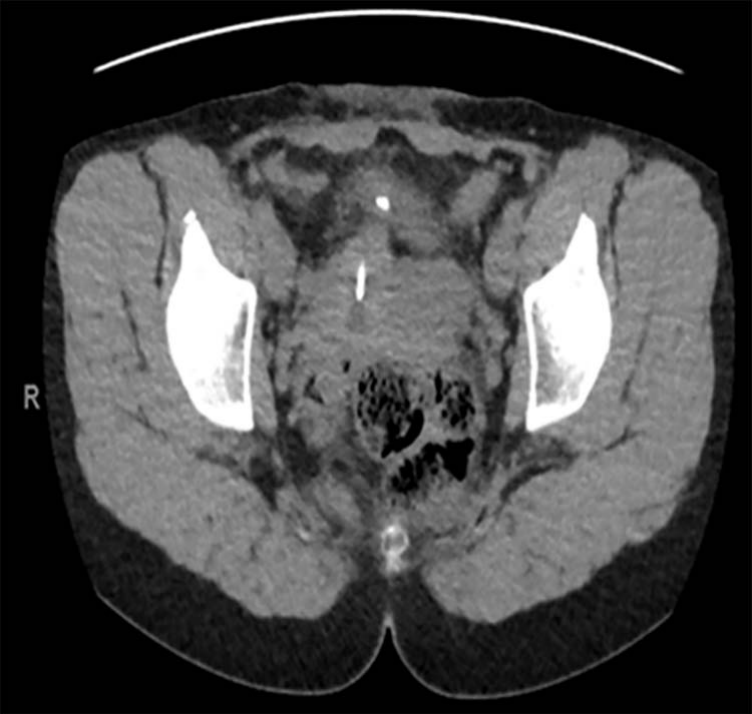
Axial CT urinary tract image demonstrating the device lying between uterus and bladder.

**Figure 4:**
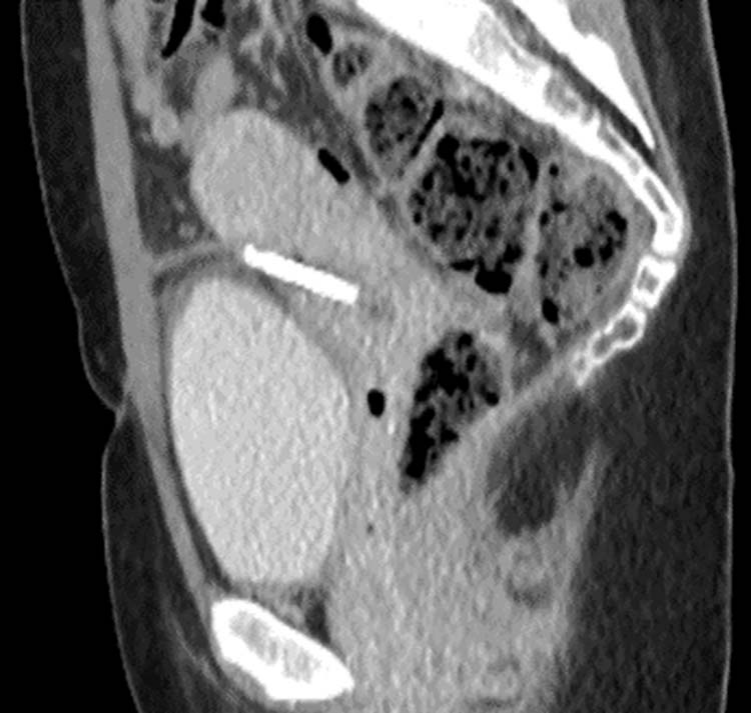
Sagittal CT urinary tract image demonstrating the device lying between uterus and bladder.

Computed tomography (CT) scan including a urographic phase revealed an IUCD in the vesico-uterine space, with one horn embedded into the bladder (Figs 2–4).

The patient was offered a transperitoneal surgical removal of the foreign body by a robotic-assisted laparoscopic approach.

### Procedure

The robotic system used was the da Vinci Surgical System (Intuitive Surgical Inc., Sunnydale, CA, USA).

Prophylactic intravenous gentamicin was administered at the beginning of the procedure. Bimanual pelvic examination under anaesthetic was unremarkable. A urinary catheter was introduced into the bladder.

Port placement was achieved in the supine position, with initial intraperitoneal access and pneumoperitoneum via the Veress method. The 12 mm camera port was sited 2 cm superior to the umbilicus and five further ports were placed under direct vision, in a similar orientation to the departmental standard approach for robotic-assisted laparoscopic prostatectomy.

Following this, the patient was put into the Trendelenburg position, and the robotic system was docked.

**Figure 5:**
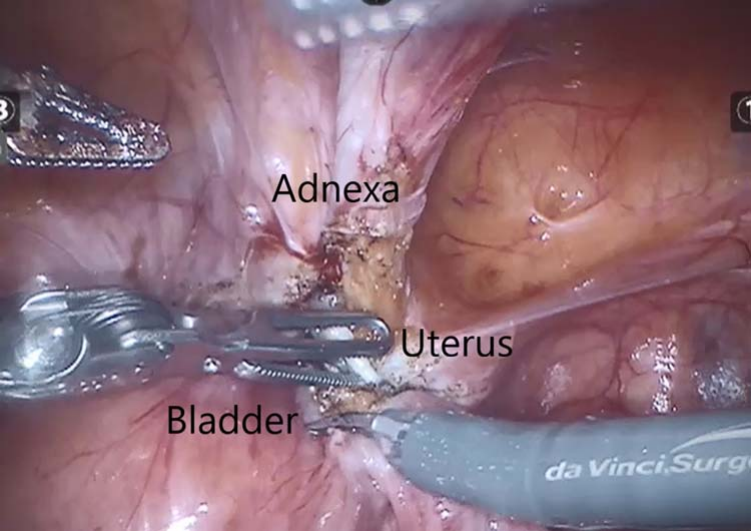
Intraoperative image demonstrating the IUCD (white) located between the bladder and uterus.

**Figure 6:**
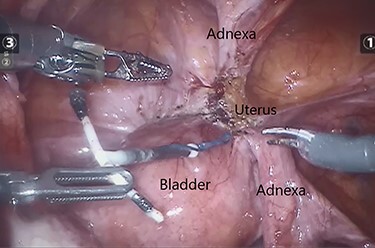
Intraoperative image demonstrating the dissected and released IUCD.

The IUCD was located in the vesico-uterine pouch, adherent to both the bladder and uterus (Fig. 5). Following careful manipulation and dissection, the IUCD was grasped and removed without complication (Fig. 6). The small bladder defect left by the device was closed with two layers of 3-0 Vicryl^™^. There was no uterine perforation.

Following a low pneumoperitoneum pressure check for haemostasis, the robotic cart was undocked. Fascial closure of port sites over 10 mm was secured with 3-0 Vicryl^™^.

### Outcome

The patient was discharged the following day through our trusted Enhanced Recovery after Surgery protocol with an indwelling catheter. The patient had a successful trial without catheter 7 days later. No cystogram was performed. No postoperative complications were reported.

## DISCUSSION

IUCDs are a common choice of contraception worldwide and account for 11% of overall contraceptive use by women in the UK [1]. Uterine perforation is a rare, but documented complication of IUCD insertion, with risk factors including breast feeding and postpartum insertion [4]. Two mechanisms of perforation have been suggested: immediate traumatic perforation and delayed perforation secondary to gradual erosion through the myometrium. Given the timespan of 7 years from insertion to presentation in our case, the latter mechanism is more likely here.

The urologist can be confronted with a migrated IUCD in several ways, with the bladder most commonly involved. Patients present with haematuria, dysuria, suprapubic discomfort or recurrent urinary tract infections. These symptoms result may from direct tissue injury or due to secondary bladder calculus formation [5]. Diagnosis is confirmed with CT scanning or equivalent cross-sectional imaging.

Management is dictated by the anatomical position of the device. Cystoscopic management is an option when the device has been determined to be entirely within the bladder lumen [6]. Laparoscopic approaches have been used to address devices embedded in the bladder wall or within the peritoneal cavity; in one review of 129 procedures for intraperitoneal migrated IUCDs, 90% underwent attempted laparoscopic removal [5]. A robotic-assisted laparoscopic approach can now be added to the list of minimally invasive options.

Although more high-quality research is needed to justify widespread uptake of robotic-assisted surgery in cases such as this, current evidence looks promising. A case report on a successful robotic-assisted laparoscopic extraction of a vaginal pessary, which had migrated into the bladder, has similarly demonstrated the potential of a robotic-assisted approach [7].

Furthermore, this case had the potential to involve a considerable amount of intracorporeal suturing in repair of the bladder and uterus. The increased dexterity afforded by a robotic-assisted approach over conventional laparoscopy for such tasks [8] was an important factor in preoperative planning. A postoperative cystogram was not felt to be necessary, given the small size of the bladder defect, but the team acknowledges that this is an important consideration in the context of bladder repair.

At the time of writing, there are no comparative studies available for IUCD removal between conventional laparoscopy and robot-assisted procedures. It is the opinion of the authors that there is no difference between the two approaches in managing these cases. Our approach would be familiar to any urologist specializing in robotic surgery, as patient positioning and port placement was based on that used in a prostatectomy [9]. In cases such as these, offering the most appropriate procedure to the patient is paramount and in a unit such as ours, where surgical and theatre staff perform robotic-assisted procedures more frequently than conventional laparoscopy, it is reasonable to proceed with the former. Of course, cost remains an important argument against choosing a robotic approach. However, with the upcoming expiry of market leader Intuitive’s earliest patents and emergence of competitors such as CMR Surgical (Cambridge, UK), costs for health providers are likely to decrease [10].

## CONCLUSION

In summary, to our knowledge, this is the first case to demonstrate that robotic-assisted laparoscopic retrieval of a migrated IUCD involving the bladder is a safe and effective management strategy.
